# Comprehensive Pharmacokinetics of the Marine-Derived PDE4 Inhibitor LY104 and Its Major Metabolite M1 in Rats: A Validated LC-MS/MS Method with Sex Comparison, Multiple-Dose, Protein Binding, Metabolic Stability, and Excretion Studies

**DOI:** 10.3390/md24060215

**Published:** 2026-06-15

**Authors:** Xiaochen Niu, Jun Zhao, Deqi Ding, Wei He, Guanhua Du, Jiejie Hao, Jianchun Zhao

**Affiliations:** 1 Marine Biomedical Research Institute of Qingdao, Ocean University of China, Qingdao 266073, China; 2 School of Medicine and Pharmacy, Ocean University of China, Qingdao 266003, China

**Keywords:** LY104, phosphodiesterase 4 inhibitor, LC-MS/MS, pharmacokinetics, sex difference, metabolic stability, excretion

## Abstract

LY104 (previously designated as B7) is a selective phosphodiesterase 4 inhibitor with promising activity against chronic obstructive pulmonary disease. We previously reported its single-dose pharmacokinetics and tissue distribution in rats. In the present study, a liquid chromatography-tandem mass spectrometry (LC-MS/MS) method was developed and validated for the simultaneous quantification of LY104 and its major metabolite M1 in rat plasma following ICH M10 guidelines. The method showed excellent linearity over 20–1200 ng/mL for both analytes, with retention times of 2.85 min (LY104) and 3.22 min (M1). Using this method, we extended our previous work in several directions. Re-analysis of previously published single-dose pharmacokinetic and tissue distribution data revealed no significant sex differences for LY104. Newly generated multiple-dose studies (1 mg/kg daily for 7 days) demonstrated no accumulation of LY104 or M1. The pharmacokinetic profile of M1 was quantified for the first time. Comprehensive in vitro investigations included plasma and liver microsomal stability, plasma protein binding, and excretion studies. This systematic preclinical pharmacokinetic characterization of LY104 and M1, incorporating re-analysis of existing data with sex stratification, newly generated multiple-dose and metabolite data, excretion studies, and comprehensive in vitro investigations, provides useful information to support further drug development and clinical trial design.

## 1. Introduction

Chronic obstructive pulmonary disease (COPD) is a progressive inflammatory respiratory disease characterized by persistent airflow limitation, representing a leading cause of morbidity and mortality worldwide [1,2]. The pathogenesis of COPD involves complex mechanisms, including oxidative stress, chronic inflammation, protease/anti-protease imbalance, and cellular senescence, driven by environmental risk factors such as cigarette smoking, air pollution, and occupational exposures [3,4,5,6,7].

Current pharmacotherapy for COPD primarily relies on bronchodilators and anti-inflammatory agents. Roflumilast is a selective phosphodiesterase 4 (PDE4) inhibitor that exerts anti-inflammatory effects by increasing cyclic adenosine monophosphate (cAMP) levels in inflammatory cells [8]. However, its clinical utility is limited by gastrointestinal adverse effects, including nausea, vomiting, and headache [9,10,11]. Doxofylline, a non-selective inhibitor with bronchodilator and anti-inflammatory activities [12,13,14], is widely used in COPD management but can cause nausea, headache, insomnia, and cardiac arrhythmias [15,16,17].

Thus, the development of novel PDE4 inhibitors with improved efficacy and safety profiles remains an urgent clinical need. In our previous study, LY104 (originally designated as B7 in our previous publication [18]), a marine-derived compound isolated from bryozoans and hydroids, was identified as a selective PDE4B inhibitor with potent anti-inflammatory efficacy in both cellular assays and in vivo models [18]. LY104 contains an ester bond structure that is readily metabolized by esterases in plasma to form its major metabolite M1. Notably, M1 exhibits a potent antispasmodic effect against histamine-induced bronchospasm, surpassing the activity of doxofylline [19]. LY104 is currently being developed for intravenous infusion for the treatment of COPD.

We have previously reported the single-dose pharmacokinetics and tissue distribution of LY104 in rats following intravenous administration [18]. However, that study did not examine potential sex differences, nor did it provide any data on the major metabolite M1. Furthermore, the multiple-dose pharmacokinetics, metabolic stability, plasma protein binding, and excretion routes of LY104 remained to be characterized.

Comprehensive pharmacokinetic studies are lacking for the majority of marine-derived bioactive molecules, despite their chemical diversity and therapeutic potential [20]. Such studies are resource-intensive, requiring significant investment, large numbers of analyses, and highly sensitive analytical methods. Therefore, establishing reliable and validated methodologies to assess the pharmacokinetics of marine-derived molecule candidates is of critical importance.

The aim of the present study was to develop and validate a sensitive LC-MS/MS method for the simultaneous quantification of LY104 and its major metabolite M1 in rat plasma, and to comprehensively characterize their preclinical pharmacokinetics in rats, including sex comparison, multiple-dose pharmacokinetics, tissue distribution, plasma protein binding, metabolic stability, and excretion.

## 2. Results and Discussion

### 2.1. LC-MS/MS Method Development

**Figure 1:**
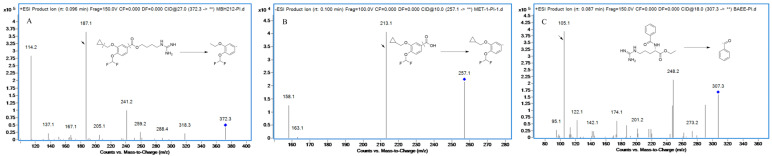
Representative product ion mass spectra of LY104 (**A**), its metabolite M1 (**B**), and BAEE (**C**).

The mass spectrometric conditions were optimized to achieve optimal sensitivity and specificity for the detection of LY104, its major metabolite M1, and the internal standard (IS) BAEE. LY104 and BAEE exhibited higher responses in positive ionization mode, which was attributed to the presence of guanidine groups in their chemical structures that readily accept protons. In contrast, M1 showed stronger and more stable signals in negative ionization mode, consistent with its acidic functional groups. Full-scan mass spectra and product ion spectra were acquired to select the most abundant and specific transitions for multiple reaction monitoring (MRM). The following precursor-to-product ion transitions were selected: *m*/*z* 372.3 → 187.1 for LY104, *m*/*z* 257.1 → 213.1 for M1, and *m*/*z* 307.3 → 105.1 for BAEE. Representative product ion spectra of LY104, M1, and BAEE are shown in Figure 1.

### 2.2. Method Validation

#### 2.2.1. Plasma Method Validation

1. Selectivity

**Figure 2:**
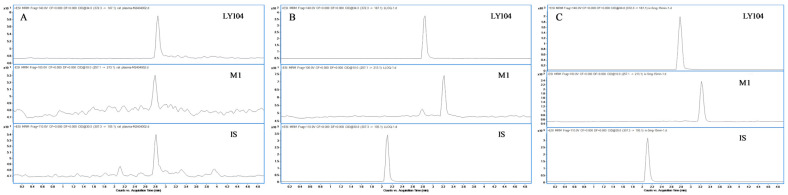
Representative MRM chromatograms of LY104, M1, and BAEE in rat plasma: (**A**) blank rat plasma; (**B**) blank rat plasma spiked with LY104, M1, and BAEE at a lower limit of quantitation (LLOQ); (**C**) rat plasma samples collected at 15 min after intravenous administration of LY104 (1 mg/kg).

Representative MRM chromatograms are shown in Figure 2. The retention times of LY104, M1, and BAEE were 2.85, 3.22, and 2.11 min, respectively. No significant peaks were observed at the retention times of the analytes in blank plasma, demonstrating adequate specificity with no matrix interference from endogenous plasma components.

2. Standard Curve and LLOQ

Calibration curves for LY104 and M1 were constructed using weighted linear least-squares regression (w = 1/x^2^) over the concentration range of 20–1200 ng/mL. The representative regression equations and correlation coefficients are presented in Table S1. The LLOQ was established at 20 ng/mL for both analytes, with all signal-to-noise ratios greater than 10.

3. Precision and Accuracy

The intra-day and inter-day precision and accuracy for LY104 and M1 are summarized in Table 1. For the low-, medium-, and high-quality control (LQC, MQC, HQC) samples, the intra-day and inter-day relative standard deviations (RSDs) ranged from 1.46% to 12.39% for both analytes. At the LLOQ, the RSDs were 7.44–9.34% for LY104 and 9.00–16.72% for M1, respectively. The relative errors (REs) for accuracy at all QC levels ranged from −7.09% to 11.90%. These results demonstrate that the developed method is reproducible and accurate for the simultaneous quantification of LY104 and M1 in rat plasma.

4. Dilution Integrity

Dilution integrity was evaluated to ensure accurate quantification of samples with concentrations exceeding the upper limit of quantification (ULOQ). Rat plasma samples spiked with LY104 and M1 at concentrations above the ULOQ were diluted 100-fold with blank rat plasma and analyzed. As shown in Table 2, the RSDs and REs for the diluted samples were ≤12.20%, demonstrating that the dilution procedure did not compromise accuracy or precision. These results confirm that samples containing LY104 and M1 at concentrations above the ULOQ can be reliably quantified after appropriate dilution.

5. Extraction Recovery

The extraction recoveries of LY104, M1, and BAEE are presented in Table 3. For LY104, the mean extraction recoveries at three QC levels ranged from 63.17% to 83.35%, with the RSDs ≤ 5.73%. For M1, the mean extraction recoveries ranged from 85.66% to 94.32% across the three QC levels, with the RSDs ≤ 4.75%. The mean extraction recovery rate of the IS was 98.63%, with the RSD ≤ 2.23%. No concentration-dependence effect on recovery was observed for either the analytes or the IS, indicating that the sample preparation procedure was consistent and reliable across the tested concentration range.

6. Matrix Effect

The matrix effect for LY104 and M1 was evaluated by analyzing LQC and HQC samples prepared in six different batches of rat plasma. As summarized in Table 4, the RSDs for LY104 were below 6.73%, with REs ranging from −2.47% to −2.48%. For M1, the RSDs were below 5.89%, and the REs ranged from −6.72% to −2.08%. These results demonstrate that no significant matrix enhancement or suppression was observed, confirming that the method is reliable for the quantification of LY104 and M1 in rat plasma samples.

7. Carryover Effect

Carryover was assessed by injecting blank samples immediately after ULOQ samples. The carryover percentages for LY104 and M1 were below 1.38% and 17.46%, respectively, while that for the IS was less than 0.09%. These values were within acceptable limits, indicating that carryover did not adversely affect the accuracy and reliability of the method.

8. Stability

The stability of LY104 and M1 in rat plasma was evaluated under various storage and processing conditions, with the results summarized in Table 5. All stability test results met the acceptance criteria, demonstrating that LY104 and M1 remained stable throughout sample processing, storage, and analysis. These findings confirm that the method is suitable for the reliable quantification of LY104 and M1 in rat plasma.

9. Incurred Sample Reanalysis (ISR)

A total of 636 plasma samples were analyzed in this study, of which 68 were selected for ISR. ISR samples were selected from low, medium, and high dose groups, covering concentrations from LLOQ to near ULOQ, with approximately equal representation of peak and elimination phase samples. For LY104, 61 out of 68 re-analysed samples met the acceptance criteria, corresponding to an ISR pass rate of 89.7%. For M1, 58 out of 68 samples met the acceptance criteria, corresponding to an ISR pass rate of 85.3%. All ISR results complied with the predefined acceptance criteria, confirming the reproducibility and reliability of the method for both LY104 and M1 in rat plasma.

#### 2.2.2. Tissue Sample Method Validation

1. Specificity

Liver tissue was selected as a representative matrix for method validation. As shown in Figure S1, no significant interfering peaks from endogenous components were observed at the retention times of LY104, M1, or BAEE in the blank matrix, demonstrating adequate specificity for tissue sample analysis.

2. Linearity

Calibration curves for LY104 and M1 were prepared in blank homogenates of liver and each target tissue (lung, colon, brain, kidney, muscle, and stomach) over the concentration range of 20–1200 ng/mL. Using liver matrix-derived calibration curves for quantifying analyte concentrations in other tissues, QC samples at low, medium, and high concentrations were prepared in homogenates of each target tissue and analyzed against the liver matrix calibration curve. All calibration curves exhibited good linearity, with correlation coefficients (r^2^ > 0.99). The representative regression equations for each tissue matrix are presented in Table S2.

3. Precision and Accuracy

The intra-day precision and accuracy for LY104 and M1 in tissue samples were evaluated by analyzing quality control samples at LLOQ, LQC, MQC, and HQC, with six replicates per level. The detailed results are summarized in Table S3. All RSDs and REs were within the acceptable limits according to the ICH M10 guidelines [21], demonstrating that the method is reliable and reproducible for the quantification of LY104 and M1 in tissue samples.

#### 2.2.3. Urine and Fecal Sample Method Validation

1. Specificity

The specificity of the method in urine and feces matrices was evaluated by analyzing blank samples from six individual rats and comparing them with samples spiked at LLOQ. The results are shown in Figures S2 and S3. For urine and feces samples, the interference from endogenous components at the retention times of LY104, M1, and the IS was within acceptable limits (≤ 20%).

2. Linearity

Calibration curves for LY104 were constructed in blank rat feces and urine matrices over the concentration range of 20–1200 ng/mL. As summarized in Table S4, all calibration curves exhibited good linearity in both matrices, with correlation coefficients (r^2^ > 0.99).

3. Precision and accuracy

The intra-day precision and accuracy for LY104 and M1 in rat feces and urine matrices were evaluated using quality control samples at low, medium, and high concentrations. The detailed results are summarized in Table S5. For LY104, the RSDs in both matrices were ≤14.28%, and REs ranged from −12.05% to 11.03%, meeting the predefined acceptance criteria. For M1, the precision in both matrices was below 14.41%, and the accuracy ranged from 0.68% to 17.57%. For M1, the upper limit of accuracy in feces was 17.57%, exceeding the commonly accepted ±15% criterion. Therefore, fecal M1 concentrations should be interpreted qualitatively rather than quantitatively. The conclusions regarding M1 fecal excretion (2.04% of dose) are presented with this limitation in mind.

### 2.3. Pharmacokinetic Studies

#### 2.3.1. Single-Dose Administration of LY104

**Figure 3:**
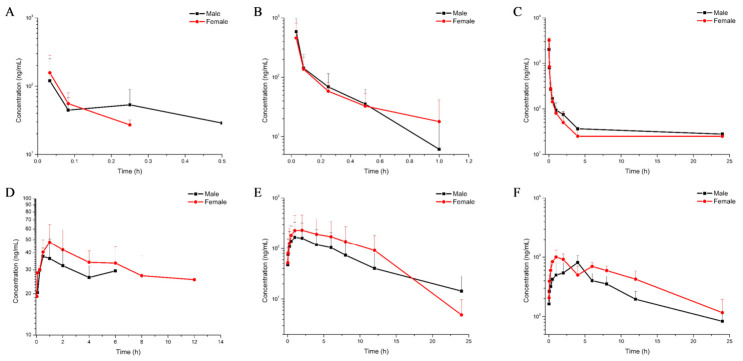
Mean plasma concentration–time profiles of LY104 (**A**–**C**) and M1 (**D**–**F**) in male and female rats after single i.v. administration of LY104 at 0.2, 1, and 5 mg/kg. Data are plotted on a semi-logarithmic scale. Error bars represent SD (*n* = 12 per group, except 5 mg/kg: *n* = 8). LY104 data are re-plotted from [18] with sex stratification; M1 data are first reported.

The single-dose pharmacokinetic parameters of LY104 in male and female rats are presented in Table S6. The raw data for LY104 were obtained from our previously published study [18] and re-analysed for sex stratification. The pharmacokinetic parameters of M1 (Table 6) are reported here for the first time as newly generated data. The mean plasma concentration-time profiles of LY104 and M1 are presented in Figure 3 on a semi-logarithmic scale. Statistical analysis (Student’s *t*-test on log-transformed AUC and C_max_ values) revealed no statistically significant sex differences for LY104 in any of the pharmacokinetic parameters evaluated (all *p* > 0.05). In contrast, sex-related differences were observed for the metabolite M1 at various doses (Table S6). However, we acknowledge that this conclusion is limited by the sample size (*n* = 6 per sex per dose group) and the lack of correction for multiple comparisons in exploratory analyses.

In addition, plasma samples from the single-dose study were re-analysed using the newly developed LC-MS/MS method to quantify, for the first time, the pharmacokinetics of the metabolite M1. The pharmacokinetic parameters of M1 are summarized in Table 6. Compared to LY104, M1 appeared more slowly in plasma with T_max_ ranging from 1.83 to 2.29 h and exhibited a longer elimination half-life (t_1/2_) of 4.90 to 8.24 h.

An important finding of this study is the absence of sex differences in the single-dose pharmacokinetics of LY104 (Table S6). In contrast to LY104, sex-related differences were observed for M1 following single intravenous administration of low (0.2 mg/kg), medium (1 mg/kg), and high (5 mg/kg) doses of LY104 (Table S6). Specifically: At the low dose, significant sex differences were noted in AUC_0–t_, CL, Vz, and MRT_0–t_ of M1; At the medium dose, sex differences were observed in AUC, CL, Vz, and C_max_ of M1; At the high dose, a sex difference was observed in AUC and CL of M1. Consistent with the previously reported linear pharmacokinetics of LY104 [18], the exposure of M1 (AUC and C_max_) increased with dose in an approximately dose-proportional manner.

It is noteworthy that sex differences in drug pharmacokinetics are more commonly observed in rats than in other species, such as dogs or humans, due to species-dependent sexual dimorphism in drug-metabolizing enzymes and transporters. Therefore, under the present experimental conditions (intravenous administration in rats), no sex-based dose adjustment would be indicated for LY104. However, confirmatory studies in higher species (e.g., dogs or non-human primates) would be warranted to fully support this conclusion.

The sex-stratified pharmacokinetic analyses were exploratory, and no correction for multiple comparisons was applied for comparisons involving multiple parameters. Therefore, the reported *p* values should be interpreted with caution.

#### 2.3.2. Multiple-Dose Administration of LY104

**Figure 4:**
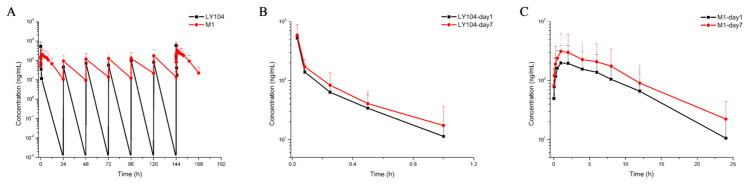
Mean plasma concentration-time profiles of LY104 and M1 in rats after seven daily intravenous doses of LY104 (1 mg/kg) (*n* = 12). Data are plotted on a semi-logarithmic scale. (**A**) Profiles from Day 1 to Day 7; (**B**) LY104 comparison; (**C**) M1 comparison.

Plasma concentrations of LY104 and its metabolite M1 were determined in rats following seven consecutive daily intravenous administrations of LY104 at 1 mg/kg. The mean plasma concentration-time profiles on Day 1 (first dose) to Day 7 (last dose) are presented in Figure 4 on a semi-logarithmic scale. The concentration-time curve of LY104 on Day 7 was similar to that observed after single-dose administration, while M1 exhibited a slightly different profile, suggesting potential metabolite accumulation.

Accumulation ratios were calculated to assess the extent of drug accumulation after repeated dosing. As summarized in Table 7, the accumulation factor for LY104 was 1.14 ± 0.62, indicating no significant accumulation in vivo. For M1, the accumulation factor was 1.73 ± 0.50, suggesting mild accumulation over seven daily doses. This moderate increase in M1 exposure should be considered in the context of repeated dosing.

These findings, reported here for the first time, demonstrate that repeated administration of LY104 does not lead to substantial accumulation of either the parent drug or its metabolite M1 in rats.

### 2.4. Tissue Distribution

**Figure 5:**
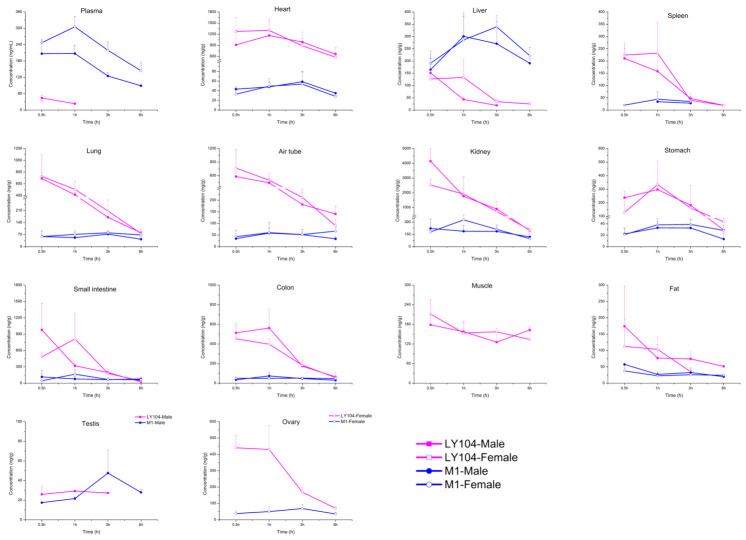
Tissue concentrations of LY104 and its metabolite M1 in male and female rats at different time points after a single intravenous dose of LY104 (1 mg/kg) (*n* = 3). LY104 data at 0.5, 1, and 3 h are from our previous study [18] and have been re-plotted with sex stratification; LY104 data at 6 h and all M1 data are reported here for the first time. Solid symbols: males; open symbols: females. Magenta: LY104; Blue: M1.

The tissue distribution of LY104 in male and female rats is presented in Figure 5. LY104 data at 0.5, 1, and 3 h are from our previously published study [18] and have been re-analysed with sex stratification. Tissue concentrations at 6 h post-dose, as well as all M1 tissue distribution data, are reported here for the first time.

**Figure 6:**
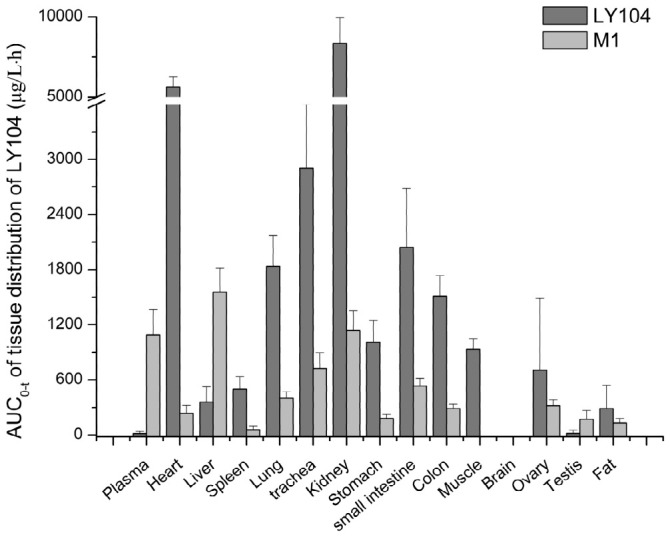
AUC_0–t_ values of LY104 and M1 in various tissues after a single i.v. dose of LY104 (1 mg/kg). Data are mean ± SD (*n* = 3 per time point, AUC_0–t_ calculated from 0.5 to 6 h).

The validated LC-MS/MS method was also successfully used to determine the tissue distribution of LY104 and its metabolites M1 after intravenous administration. The mean concentration and distribution trends of LY104 and its metabolite M1 in rat tissues after intravenous administration (1 mg/kg) are shown in Figure 5. LY104 and its metabolite M1 were detected in all examined tissues as early as 30 min post-dose, with the highest concentrations observed in the heart and kidney (Figure 5 and Table S8). Peak tissue concentrations were generally achieved at 1 h post-dose. By 6 h post-dose, tissue concentrations had declined but remained detectable in most tissues. No statistically significant differences under the current experimental conditions were observed between male and female rats in tissue concentrations of LY104 at 0.5, 1, 3, or 6 h, consistent with the absence of sex differences in plasma pharmacokinetics (Section 2.3). Tissue concentrations of both LY104 and M1 declined progressively after 6 h, with no accumulation in the body. Higher concentrations of LY104 and M1 were detected in the kidneys, which is consistent with the kidneys being involved in the excretion of these compounds. However, tissue concentration alone does not prove that the kidneys are the primary excretory organs; direct measurement of urinary excretion (Section 2.8) provides complementary evidence. Higher levels of M1 were detected in plasma and the liver, which is consistent with these being sites of LY104 metabolism, but definitive demonstration would require enzyme activity assays or metabolite formation kinetics. LY104 and M1 were widely distributed in all examined tissues except the brain. The target organs, lungs and trachea have higher levels of LY104 and M1. Except in plasma, liver, and testes (where M1 was higher), LY104 exhibited significantly higher tissue concentrations and AUC values than M1 in all other tissues (Figure 6). LY104 achieved higher total tissue concentrations than M1 in most organs, suggesting that the parent compound may be the primary contributor to pharmacological activity. However, the free (unbound) concentration, intrinsic potency, and target-site exposure of M1 also need to be considered. The relative contributions of LY104 and M1 to in vivo efficacy require further investigation using appropriate PK/PD modelling.

Following intravenous administration of LY104 at 1 mg/kg, the peak lung concentration of LY104 reached 737.94 ng/mL (approximately 1.99 μM). This value exceeds the IC_50_ of LY104 for PDE4B inhibition (0.90 μM) [18] by approximately 2.2-fold, indicating that LY104 achieves pharmacologically relevant concentrations in the target organ (lung).

BBB permeability is a critical determinant of potential CNS effects. For drugs intended to act peripherally, limited BBB penetration is desirable to avoid CNS-related adverse effects. In the present study, we systematically evaluated the distribution of LY104 and its metabolite M1 in brain tissue. No LY104 was detected in brain at 0.5, 1, 3, and 6 h (Table S8). The metabolite M1 was barely detectable in brain tissue at very low levels, with concentrations consistently below 20 ng/g at all time points (Table S9). This negligible brain penetration addresses a key limitation of existing PDE4 inhibitors and supports a favorable safety profile for LY104.

The distribution profile of LY104 offers a distinct advantage for COPD treatment. While many PDE4 inhibitors suffer from dose-limiting emetic side effects due to CNS penetration, LY104 demonstrated negligible brain accumulation. Concurrently, LY104 showed relatively higher distribution to the lungs (Kp = 118.36) and trachea (Kp = 187.36) compared with brain tissue (Table S10), indicating accumulation in these tissues under the current experimental conditions. This favorable profile can be attributed to the structural features of LY104: the terminal guanidine group reduces blood–brain barrier permeability, thereby mitigating CNS-related adverse effects; additionally, the presence of aromatic hydrophobic groups and the guanidine moiety imparts a cationic amphiphilic character, resulting in relatively higher lung exposure compared with brain. However, LY104 also showed substantial exposure in non-target organs, particularly the kidney and heart, indicating that the compound is not exclusively distributed to the respiratory tract. For a PDE4 inhibitor intended to treat respiratory diseases, this profile enhances local drug concentrations at the site of action while minimizing systemic and CNS toxicity, representing an improvement over existing agents such as roflumilast.

The sex-stratified tissue distribution analyses were exploratory in nature, given the small sample size (*n* = 3 per sex per time point) and the lack of correction for multiple comparisons. These isolated significant findings should be interpreted with caution.

### 2.5. Blood-Plasma-Partitioning of LY104

The blood-to-plasma partition (B/P) ratio of LY104 in rat blood was evaluated at three concentrations (200, 1000, and 5000 ng/mL). The mean partition ratio was 0.60 ± 0.03, with no apparent concentration dependence (Table 8). The concentration ratio of whole blood to plasma was 0.84 ± 0.21 and the ratio of blood cells to plasma was 0.10 ± 0.07. These results indicate that LY104 is predominantly distributed in the plasma fraction rather than associating with blood cells. Therefore, the use of plasma rather than whole blood for sample analysis is appropriate and accurately reflects the systemic drug concentration in rats.

### 2.6. Plasma Protein Binding

Plasma protein binding of LY104 was evaluated in mouse, rat, and dog plasma, as well as in human serum albumin (HSA), using an ultrafiltration method coupled with LC-MS/MS analysis. Three concentrations were selected based on the expected plasma concentration range of LY104. As summarized in Table 9, the protein binding of LY104 exceeded 99% in all tested matrices. No significant concentration-dependent variations were observed within the same species, indicating that protein binding was independent of concentration over the tested range. Furthermore, the protein binding rates were comparable across all species tested, demonstrating no significant species difference in the plasma protein binding of LY104.

### 2.7. In Vitro Stability Studies

#### 2.7.1. Plasma Stability

The metabolic stability of LY104 was evaluated in rat, dog, and human plasma, with results shown in Table 10. Marked species-dependent differences were observed. LY104 was rapidly and completely converted to M1 in rat and human plasma. In rat plasma, LY104 exhibited a t_1/2_ of 0.72 ± 0.03 min and an intrinsic clearance of 1927.48 ± 82.87 μL/min/mg. In human plasma, similar rapid metabolism was observed, with a t_1/2_ of 0.69 ± 0.04 min and a clearance of 2021.57 ± 107.02 μL/min/mg. In contrast, LY104 was metabolized much more slowly in dog plasma, with a half-life of 123.39 ± 93.70 min and a clearance of 23.30 ± 25.62 μL/min/mg.

**Figure 7:**
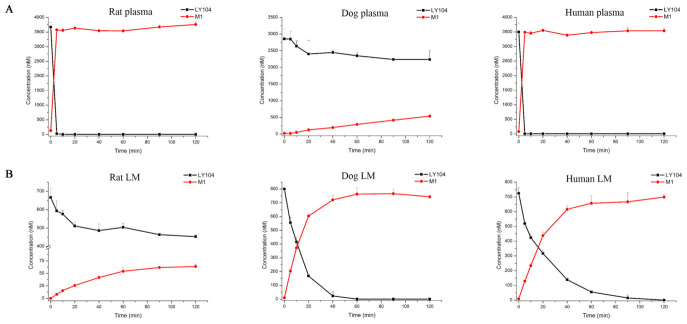
In vitro metabolic stability of LY104 in plasma (**A**) and liver microsomes (**B**) from rat, dog, and human (*n* = 3).

In rat and human plasma, LY104 is rapidly hydrolyzed via ester bond cleavage to form its metabolite M1. The conversions were nearly complete, with molar formation ratios (LY104 to M1) approaching 1:1 (Figure 7). In dog plasma, however, the formation of M1 was substantially lower, with a molar ratio of approximately 1:0.2.

The metabolism of LY104 in human plasma is extremely rapid, with immediate conversion to M1 upon contact with plasma. Therefore, special attention should be paid to sample handling and processing when working with human plasma to ensure accurate quantification of the parent drug. The similar plasma metabolic stability of LY104 in rat and human plasma suggests that the rat may be a useful model for further pharmacokinetic investigations, but cross-species extrapolation should be made with caution. The observed species-dependent metabolism of LY104 in plasma is consistent with known species differences in carboxylesterase activity [22,23]: high esterase levels in rat and human plasma promote rapid hydrolysis of LY104, while low esterase levels in dog plasma result in slower metabolism. These findings highlight the importance of selecting appropriate animal models for preclinical pharmacokinetic studies of ester-containing drugs, with rat plasma closely resembling human plasma in terms of LY104 metabolic behavior.

#### 2.7.2. Liver Microsomal Stability

The metabolic stability of LY104 was evaluated in liver microsomes from three species (rat, dog, and human), with results shown in Table 10 and Figure 7. Species-dependent differences in metabolic rate were observed. In rat liver microsomes, LY104 exhibited a t_1/2_ of 40.44 ± 4.20 min and an intrinsic clearance of 34.53 ± 3.81 μL/min/mg. In human liver microsomes, the t_1/2_ was 14.28 ± 2.35 min with a clearance of 98.92 ± 16.82 μL/min/mg. In dog liver microsomes, LY104 was metabolized most rapidly, with a t_1/2_ of 8.97 ± 1.09 min and a clearance of 155.93 ± 17.64 μL/min/mg.

The rank order of liver microsomal metabolic rate across species was dog > human > rat. The conversion of LY104 to its major metabolite M1 was nearly complete (approaching 1:1) in dog and human liver microsomes. In rat microsomes, the conversion was substantially lower (approximately 1:0.1), indicating a marked species difference in metabolic stability. This pattern differs from that observed in plasma stability studies. These findings suggest that the enzymes responsible for LY104 metabolism in liver microsomes differ from those in plasma, and highlight the importance of evaluating both hepatic and extrahepatic metabolic pathways when characterizing the overall disposition of LY104.

### 2.8. Excretion Studies

**Figure 8:**
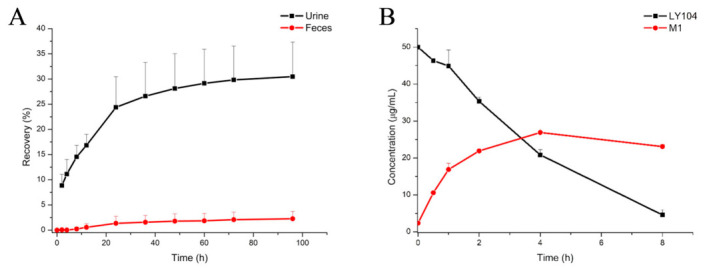
Excretion studies of LY104. (**A**) Cumulative excretion profiles in urine and feces over 96 h after i.v. administration of LY104 (1 mg/kg). (**B**) Conversion of LY104 to M1 by rat fecal flora at 37 °C. Data are mean ± SD (*n* = 6 for (**A**); *n* = 3 for (**B**)).

The excretion of LY104 and its metabolite M1 in urine and feces was investigated following a single intravenous administration of LY104 at 1 mg/kg to rats. Cumulative excretion was monitored over 96 h post-dose using the validated LC-MS/MS method, and results are shown in Figure 8.

In urine, both LY104 and M1 were detected, with mean cumulative excretion rates of 14.38% and 16.09% of the administered dose, respectively. In contrast, negligible amounts were recovered in feces, with mean cumulative excretion rates of only 0.22% for LY104 and 2.04% for M1. The total recovery of LY104 and M1 in urine and feces combined was 32.73% of the administered dose. This indicates that the majority of the dose is not accounted for as LY104 or M1, suggesting extensive biotransformation into additional, unquantified metabolites. Metabolite profiling and improved mass balance are needed to fully characterise the elimination pathways of LY104. In vitro incubation with rat fecal flora showed that gut microbiota can convert LY104 to M1 (≈50% conversion, Figure 8). However, given the intravenous route of administration and negligible fecal recovery of LY104, the in vivo relevance of this finding remains unclear. The contribution of intestinal flora to LY104 disposition may be limited under the current dosing conditions.

Sex-related differences in excretion were also evaluated. No significant differences between male and female rats were observed in the cumulative urinary or fecal excretion of either LY104 or M1 (*p* > 0.05), indicating that the elimination pathways of LY104 are not sex-dependent.

### 2.9. Limitations

Several limitations should be acknowledged. The single-dose pharmacokinetic and tissue distribution data for LY104 at 0.5, 1, and 3 h were originally published in our previous study [18] and have been re-analysed here with sex stratification. The specific hepatic enzymes responsible for LY104 metabolism were not identified; enzyme phenotyping studies are warranted. PBPK modeling using the generated preclinical data was not performed but represents an important future direction. The study includes numerous comparisons across sex, dose, time point, tissue, analyte, and pharmacokinetic parameter, and no correction for multiple comparisons was applied for exploratory analyses (e.g., tissue distribution, multiple PK parameters); therefore, isolated statistically significant findings should be interpreted with caution. The small sample size (*n* = 3 per sex per time point for tissue distribution) limits the ability to definitively conclude absence of sex-dependent distribution. Although the intravenous administration used in this study aligns with the intended clinical route (intravenous infusion), direct extrapolation from rats to humans is not straightforward. Cross-species differences in drug metabolism and disposition must be considered. Therefore, these findings should be viewed as preclinical characterisation requiring validation in human studies. The conclusion that LY104 plays the major pharmacological role in vivo is based primarily on total tissue concentrations. Free (unbound) concentrations in target tissues and comparative PK/PD modelling were not performed, limiting the strength of this conclusion. The plasma protein binding of M1 was not determined in this study, which is a limitation given that M1 is the major active metabolite. Human protein binding was assessed using HSA rather than pooled human plasma due to the instability of LY104 in human plasma. However, the absence of other plasma binding proteins may affect generalizability. Low or undetectable brain concentrations indicate limited blood–brain barrier penetration under the current experimental conditions, but definitive proof would require additional studies (e.g., in situ brain perfusion or formal BBB permeability assays). Only 32.73% of the administered dose was recovered as LY104 and M1 in urine and feces. The majority of the dose remains unaccounted for, and no metabolite profiling was performed to identify other potential metabolites. This limits the comprehensiveness of the ADME characterisation.

## 3. Materials and Methods

### 3.1. Chemicals and Reagents

LY104 (purity > 99%) and its metabolite M1 (purity > 99%) were kindly provided by Prof. Jiejie Hao from Ocean University of China. BAEE (N-benzoyl-L-arginine ethyl ester hydrochloride), used as the IS, was purchased from Shanghai Aladdin Biochemical Technology Co., Ltd. (Shanghai, China). BAEE was selected because it shares structural features with LY104 (guanidine group, benzene ring, ester bond) and demonstrated consistent recovery (98.63%), no matrix effect, and no mutual interference. Acetonitrile and methanol (all LC-MS grade) were purchased from Adamas-beta (Shanghai, China), and formic acid (LC-MS grade) was obtained from Thermo Fisher Scientific (Shanghai, China).

Mouse, rat, and dog plasma were purchased from Guangzhou Hongquan Biotechnology Co., Ltd. (Guangzhou, China). Human plasma was obtained from healthy volunteers. Human serum albumin and NADPH-Na_4_ were obtained from Beijing Solarbio Science & Technology Co., Ltd. (Beijing, China). Liver microsomes from all three species (rat, dog, and human) were purchased from Wuhan Pulite Biomedical Technology Co., Ltd. (Wuhan, China).

### 3.2. Chromatography Mass Spectrometry Conditions

Chromatographic separation was performed on an Agilent 1290 HPLC system (Agilent Technologies, Waldbronn, Germany) equipped with a Capcell Pak C18 column (2.0 × 50 mm, 5 μm). The column temperature was maintained at 30 °C. The mobile phase consisted of water containing 0.1% (*v*/*v*) formic acid (solvent A) and acetonitrile containing 0.1% (*v*/*v*) formic acid (solvent B), with a gradient elution program as follows: 0–0.5 min, 5% B; 0.5–2.0 min, 5–35% B; 2.0–3.0 min, 35–95% B; 3.0–3.5 min, 95% B; 3.5–3.8 min, 95–5% B; 3.8–5.0 min, 5% B. The flow rate of the mobile phase was 0.4 mL/min, and the injection volume was 5 μL.

The HPLC system was coupled to an Agilent 6460 triple quadrupole mass spectrometer (Agilent Technologies, Waldbronn, Germany) equipped with an electrospray ionization (ESI) source. Detection was performed in multiple reaction monitoring (MRM) mode. LY104 and BAEE were ionized in positive ionization mode, while M1 was analyzed in negative ionization mode. The optimized ion source parameters were as follows: gas temperature, 350 °C; gas flow rate, 11 L/min; nebulizer pressure, 30 psi; capillary voltage, 4 kV. The fragmentor voltages were optimized at 140 V for LY104, 100 V for M1, and 110 V for BAEE. Collision energies were set at 34 V for LY104, 10 V for M1, and 30 V for BAEE. The following precursor-to-product ion transitions were used for quantification: *m*/*z* 372.1 → 187.1 for LY104, *m*/*z* 257.1 → 213.1 for M1, and *m*/*z* 307.3 → 105.1 for BAEE.

### 3.3. Preparation of Stock Working Solutions

Stock solutions of LY104 and M1 were prepared by accurately weighing each compound and dissolving in dimethyl sulfoxide (DMSO) to a final concentration of 10 mg/mL. Equal volumes of the individual stock solutions were then mixed to obtain a combined stock solution containing both analytes. A series of working solutions for calibration curves were prepared by serial dilution of the combined stock solution with 60% acetonitrile in water, yielding concentrations ranging from 200 to 12,000 ng/mL. Quality control (QC) working solutions were similarly prepared at three concentration levels: LQC, MQC, and HQC. The internal standard BAEE was prepared as a 10 mg/mL stock solution in DMSO and subsequently diluted with acetonitrile to a working concentration of 50 ng/mL. The stock solutions were stored at −20 °C until use. All working solutions were stored at 4 °C until use.

### 3.4. Preparation of Calibration Standards and QC Samples

Calibration standards and QC samples were prepared by spiking 5 μL of the appropriate working solutions (10-fold concentration) into 50 μL of blank rat matrix (plasma, tissue, feces, or urine). The mixture was precipitated with 100 μL of acetonitrile containing the internal standard BAEE (50 ng/mL), vortex-mixed thoroughly for 1 min, and centrifuged at 14,000 rpm for 10 min at 4 °C. An aliquot of 100 μL of the supernatant was pipetted into autosampler vials, and 5 μL was injected into the LC-MS/MS system for analysis.

### 3.5. Animals

All animal experiments were approved by the Animal Experiment Ethics Committee of Marine Biomedical Research Institute of Qingdao (Approval No.: E-MBPT-2024-4-12, 12 April 2024, Qingdao, China). Male and female Wistar rats (weighing 180–220 g, 6–8 weeks old) were purchased from Jinan Pengyue Experimental Animal Breeding Co., Ltd. (Jinan, China). Animals were housed in specific pathogen-free (SPF) facilities under controlled environmental conditions: temperature of 24 ± 2 °C, relative humidity of 60 ± 5 and a 12 h light/dark cycle. Food and water were provided *ad libitum*. Animals were acclimatized for at least 7 days prior to the experiments. Rats were euthanized by intraperitoneal injection of sodium pentobarbital (150 mg/kg).

### 3.6. Sample Preparation

Plasma samples: 150 μL blood samples were collected from the orbital sinus at designated time points after administration and transferred to heparinized tubes. Plasma was separated by centrifugation at 8000 rpm for 10 min at 4 °C. An aliquot of 50 μL plasma was mixed with 5 μL 60% acetonitrile in water, followed by the addition of 100 μL of acetonitrile containing BAEE (50 ng/mL). The mixture was vortex-mixed thoroughly for 1 min and centrifuged at 14,000 rpm for 10 min at 4 °C. An aliquot of 100 μL of the supernatant was transferred to an autosampler vial for LC-MS/MS analysis.

Tissue samples: After euthanasia, the following tissues were immediately collected: heart, liver, spleen, lung, kidney, stomach, colon, small intestine, brain, fat, testes, ovaries, muscle, and trachea. Tissues were rinsed with ice-cold saline to remove residual blood. Each tissue sample was weighed and homogenized with three volumes (1:3, *w*/*v*) of ice-cold distilled water using a tissue homogenizer. The homogenates were stored at −80 °C until analysis. Tissue samples were then processed following the same protein precipitation procedure as described for plasma samples.

Feces and urine samples: Fecal samples were weighed and homogenized with three volumes (1:3, *w*/*v*) of distilled water using a tissue homogenizer. Urine samples were collected and centrifuged at 8000 rpm for 10 min at 4 °C to remove particulate matter, and the supernatant was used for analysis. Both fecal homogenates and urine supernatants were then processed following the same protein precipitation procedure as described for plasma samples.

### 3.7. Method Validation in Plasma

#### 3.7.1. Specificity

Selectivity of the method was evaluated by analyzing blank biological samples, blank matrix spiked with LY104 and M1 at LLOQ, and biological samples collected from rats after intravenous administration of LY104. Chromatograms obtained from these samples were compared to assess potential interference from endogenous components at the retention times of the analytes and the IS.

#### 3.7.2. Linearity

Calibration curves were constructed by plotting the peak area ratio of each analyte to the IS (y) against the nominal concentration (x). Calibration curves were evaluated using a weighted linear least squares regression method (w = 1/x^2^). The abscissa was the theoretical concentration of the analytes, and the ordinate was the ratio of the peak area between analytes and IS. The linearity degree was expressed by the correlation coefficient (r), and r^2^ should be greater than 0.99.

#### 3.7.3. LLOQ, Precision and Accuracy

LLOQ was defined as the lowest concentration of the calibration curve with a signal-to-noise ratio (S/N) greater than 10, and acceptable precision and accuracy.

The intra-day and inter-day precision and accuracy of the method were evaluated by analyzing six replicate QC samples at LLOQ, LQC, MQC, and HQC. Precision was expressed as the relative standard deviation (RSD, %), and accuracy was expressed as the relative errors (RE, %). The acceptance criteria were as follows: RSD ≤ 15% and RE within ±15% for all QC levels, except at the LLOQ where RSD ≤ 20% and RE within ±20% were considered acceptable.

#### 3.7.4. Dilution Integrity

To ensure accurate quantification of biological samples with concentrations exceeding the upper limit of quantification (ULOQ), dilution integrity was evaluated. Samples containing LY104 and M1 at concentrations above the ULOQ were prepared and diluted 100-fold with blank matrix. The diluted samples were processed and analyzed following the same procedure as described above. The measured concentrations were multiplied by the dilution factor and compared with the nominal concentrations. Dilution integrity was considered acceptable if RSD was ≤15% and the RE was within ±15%.

#### 3.7.5. Extraction Recovery

The extraction recoveries of LY104, M1 and BAEE were evaluated by comparing peak areas obtained from samples spiked with the analytes before extraction with those from blank matrix extracts spiked with the analytes after extraction (post-extraction spiked samples). Recovery was determined at three QC concentration levels with three replicates at each level. The recovery was calculated as the percentage of the pre-extraction peak area relative to the post-extraction peak area. Consistent and reproducible recovery with RSD ≤ 15% was considered acceptable.

#### 3.7.6. Matrix Effects

The matrix effects for LY104 and M1 were evaluated by analyzing at least 3 replicates of low and high QCs, each QC prepared using matrix from at least 6 different sources. For each individual matrix source evaluated, the RE should be within ±15% of the nominal concentration and the RSD should not be greater than 15%.

#### 3.7.7. Carry-Over Effect

The carry-over effect of LY104, M1, and BAEE was assessed by injecting blank plasma samples immediately after the ULOQ sample. The procedure was repeated six times to ensure reliability. Carry-over was considered acceptable if the peak area of each analyte (LY104 and M1) in the blank sample did not exceed 20% of the peak area at LLOQ, and the peak area of the IS did not exceed 5% of its mean peak area in all the QC samples.

#### 3.7.8. Stability Assay

The stability of LY104 and M1 was evaluated using LQC and HQC samples under various storage and handling conditions with three replicates. Bench-top stability was analyzed using the samples in rat plasma kept on ice for 20 min before processing. Post-preparative stability was determined by evaluating the processed samples kept at room temperature for 6 h prior to analysis. Long-term stability was examined by analyzing the QC samples in rat plasma stored at −80 °C for 35 days before analysis. Freeze–thaw stability was investigated by analyzing samples subjected to three freeze (−80 °C)-thaw (room temperature) cycles. Autosampler stability was determined by evaluating the processed samples kept in the autosampler at 20 °C for 24 h before injection. For all stability tests, the concentrations measured after storage were compared with those of freshly prepared samples. Stability was considered acceptable if the mean concentration at each QC level was within ±15% of the nominal concentration.

#### 3.7.9. Incurred Sample Reanalysis

Incurred sample reanalysis (ISR) was conducted to confirm the reproducibility of the method for authentic study samples. A subset of study samples was selected and re-analysed in a separate analytical run within the established stability window of the analytes. The percent difference (%) between the initial and repeat concentrations was calculated using the following equation:

(1) $$\mathrm{Difference}\;(\%)\;=\;\frac{\mathrm{repeat}\;\mathrm{value}-\mathrm{initial}\;\mathrm{value}}{\mathrm{mean}\;\mathrm{value}}\times \;100\%$$

ISR was considered acceptable if the difference between the two measurements was within ±20% for at least two-thirds of the reanalyzed samples.

### 3.8. Method Validation in Tissue and Excreta Matrices

The method was partially validated in rat liver homogenate and excreta matrices (feces and urine) according to ICH M10 guidelines [21]. The following parameters were evaluated: specificity, linearity, LLOQ, and intra-day precision and accuracy. The procedures were performed as described in Section 3.7.

### 3.9. Pharmacokinetic Study in Rats

#### 3.9.1. Single-Dose Pharmacokinetics

The single-dose pharmacokinetic study of LY104 was conducted as previously described [18]. Briefly, thirty-two Wistar rats were randomly divided into three groups, with an equal number of male and female rats in each group. The rats received a single intravenous administration of LY104 at low (0.2 mg/kg), medium (1 mg/kg), or high (5 mg/kg) doses. Blood samples (approximately 0.2 mL) were collected from orbital venous plexus at the following time points: pre-dose (0), 2, 5, 15 and 30 min, 1, 2, 4, 6, 8, 12, and 24 h post-dose. Blood samples were collected into heparinized tubes and immediately centrifuged at 8000 rpm for 10 min at 4 °C to obtain plasma.

In our previous report [18], the single-dose pharmacokinetic data were presented without sex stratification. For the present study, we re-analysed the raw data to evaluate potential sex differences in LY104 pharmacokinetics. Additionally, plasma samples from the single-dose study were re-analysed using the newly developed LC-MS/MS method (Section 2.1) to quantify, for the first time, the pharmacokinetics of the metabolite M1.

#### 3.9.2. Multiple-Dose Pharmacokinetics

The multiple-dose pharmacokinetic study was conducted using the same dose level as the medium-dose single-dose study (1 mg/kg). Rats received daily intravenous administration of LY104 at 1 mg/kg for seven consecutive days. The first dose of the multiple-dose regimen corresponded to the medium-dose group in the single-dose study [18]. Blood samples were collected at the following time points: on days 2–6, samples were collected 15 min before and 15 min after each dose; on day 7, samples were collected at pre-dose (0) and 2, 5, 15 and 30 min, 1, 2, 4, 6, 8, 12, and 24 h. All samples were stored at −80 °C until analysis. Multiple-dose samples were processed immediately after collection using the same ice-cold protein precipitation protocol as single-dose samples, ensuring consistent handling and minimizing hydrolysis.

### 3.10. Tissue Distribution Studies

Tissue distribution studies of LY104 were conducted as previously described [18]. Briefly, twenty-four Wistar rats were divided into four groups, with an equal number of male and female rats in each group. The rats were administered LY104 (1 mg/kg) via intravenous administration, and were euthanized at 0.5, 1, 3, and 6 h post-dose (*n* = 6 at each time point). Blood was collected immediately, and the following tissues were collected: heart, liver, spleen, lung, kidney, stomach, colon, small intestine, trachea, muscle, fat, brain, ovary, and testis. The removed tissue samples were rinsed with cold 0.9% normal saline, dried with filter paper, weighed. The tissues were then immediately homogenized with three volumes (1:3, *w*/*v*) of ice-cold distilled water, and the homogenates were stored at −80 °C until analysis.

In our previous report [18], tissue distribution data were presented for 0.5, 1, and 3 h post-dose without sex stratification. In the present study, we extended the observation to 6 h post-dose and re-analysed all raw data (0.5, 1, 3, and 6 h) to evaluate potential sex differences in LY104 tissue distribution. In addition, tissue samples were re-analysed using the newly developed LC-MS/MS method (Section 2.1) to quantify, for the first time, the distribution of the metabolite M1.

### 3.11. In Vitro Stability Studies

#### 3.11.1. Plasma Stability

The metabolic stability of LY104 was evaluated in rat, dog, and human plasma, respectively. LY104 was spiked into blank plasma to yield a final concentration of 3 μM. After incubation at 37 °C for 0, 5, 10, 20, 40, 60, 90, and 120 min, 100 μL of the incubation mixture was collected. The reaction was terminated by adding a two-fold volume of ice-cold acetonitrile containing BAEE (50 ng/mL) as the internal standard. Each sample was prepared and analyzed in triplicate.

#### 3.11.2. Microsomal Stability

Metabolic stability of LY104 was evaluated using liver microsomes from three species (rat, dog, and human). LY104 (1 μM) was incubated with liver microsomes (0.5 mg/mL) in phosphate buffer at 37 °C. The reaction was initiated by adding NADPH (1 mM) as a cofactor. At predetermined time points (0, 5, 10, 20, 40, 60, 90, and 120 min), 50 μL aliquots were withdrawn, and the reaction was immediately terminated by adding 100 μL of ice-cold acetonitrile containing the internal standard BAEE (50 ng/mL). All samples were vortex-mixed, centrifuged, and the supernatant was analyzed by LC-MS/MS. Each time point was assessed in triplicate.

### 3.12. Blood-to-Plasma Partitioning

The blood-to-plasma (B/P) ratio was determined to characterize the distribution profile of the drug between whole blood and plasma, which is critical for understanding in vivo drug disposition. Fresh heparinized whole blood was collected from rats and spiked with LY104 working solutions to achieve final concentrations of 200, 1000, and 5000 ng/mL. After incubation on ice for 30 min, an aliquot of whole blood was withdrawn for analysis, and the remaining whole blood was centrifuged at 8000 rpm at 4 °C for 10 min to separate plasma and red blood cell fractions. The concentrations of LY104 in whole blood, plasma, and red blood cells were determined using the validated LC-MS/MS method. The B/P ratio was calculated using the following equation:

(2) $$B/P\;\mathrm{Ratio}\;=\;\frac{H\times C_{e}+\left(1-H\right)\times C_{p}}{C_{p}}$$

where *H* is the hematocrit value (0.44 for rats [24]), *C_e_* is the concentration of LY104 in red blood cells, and *C_p_* is the concentration of LY104 in plasma. All experiments were performed in triplicate.

### 3.13. Plasma Protein Binding

The plasma protein binding (PPB) of LY104 was determined using a modified ultrafiltration technique [25] employing an Amicon^®^ ultra-0.5 device (Merck KGaA, Darmstadt, Germany) to minimize non-specific binding (NSB), as previously applied successfully [26]. To validate the suitability of this device for LY104, we performed a non-specific binding (NSB) assessment by spiking LY104 into phosphate-buffered saline (without plasma proteins) at 200, 1000, and 5000 ng/mL. The recovery of LY104 in the ultrafiltrate exceeded 95% (NSB < 5%) at all tested concentrations (*n* = 3).

The plasma protein binding (PPB) rate of LY104 was evaluated in mouse plasma, rat plasma, dog plasma, and human serum albumin. Human serum albumin was used as a simplified model system instead of pooled human plasma because LY104 is rapidly hydrolyzed by plasma esterases in human plasma (t_1/2_ = 0.69 ± 0.04 min; see Section 2.7.1), making it impossible to accurately determine the protein binding of the parent drug in full plasma. HSA provides an esterase-free environment that ensures the stability of LY104 during the incubation period. Albumin is the most abundant protein in human plasma and serves as the primary binding protein for most small molecule drugs.

Working solutions at low, medium, and high concentrations were spiked into each blank matrix to achieve final LY104 concentrations of 200, 1000, and 5000 ng/mL, respectively. After incubation on ice for 30 min, aliquots of the spiked plasma samples were transferred into ultrafiltration devices. Each concentration was prepared in triplicate. The samples were then centrifuged at 10,000 rpm at 4 °C for 10 min. 100 μL of the ultrafiltrate (containing free drug) was collected for the measurement of the free LY104 concentration (*Df*). An aliquot of 100 μL of the plasma sample (ultrafiltration residue, stored at 4 °C) was collected for the determination of the total LY104 concentration (*Dt*). The PPB rate was calculated using the following equations [27]:

(3) $$\mathrm{PPB}\;(\%)\;=\;\frac{\mathrm{Dt}-\mathrm{Df}}{\mathrm{Dt}}\times \;100\%$$

where *Dt* is the total drug concentration in plasma and *Df* is the free drug concentration in the ultrafiltrate.

Incubation was performed on ice (0–4 °C) rather than at 37 °C because LY104 is rapidly hydrolyzed by plasma esterases at 37 °C (see Section 2.7.1). Low-temperature incubation was necessary to prevent extensive metabolism of LY104 to M1 during the protein binding experiment, ensuring accurate determination of the parent drug’s protein binding.

### 3.14. In Vitro Incubation of LY104 with Fecal Flora of Rat

Fresh fecal samples were collected from rat into sterile tubes immediately after euthanasia. The feces were mixed with anaerobic broth at a ratio of 1:30 (*w*/*v*) and homogenized thoroughly. LY104 was dissolved in normal saline, and 5 μL of the resulting solution was added to 0.5 mL of the fecal homogenate. The mixture was then incubated anaerobically at 37 °C. At designated time points (0, 0.5, 1, 2, 4, and 8 h), an aliquot of the incubation mixture was added 200 μL of acetonitrile spiked with the IS. The samples were vortex mixed and centrifuged at 14,000 rpm for 10 min at 4 °C. Subsequently, 5 μL of the supernatant was injected into the LC-MS/MS system for analysis.

### 3.15. Excretion

To investigate the excretion profiles of LY104 and its metabolite M1, six Wistar rats (weighing 180 ± 20 g, half male and half female) received a single intravenous dose of LY104 at 1 mg/kg. Immediately after dosing, each rat was placed individually in a metabolic cage for the collection of urine and feces. Samples were collected at the following time intervals: 0–2, 2–4, 4–8, 8–12, 12–24, 24–36, 36–48, 48–60, 60–72, and 72–96 h post-dose. The weight of feces and volume of urine from each time interval were recorded. All samples were stored at −80 °C until analysis.

### 3.16. Statistical Analysis

Pharmacokinetic parameters, including maximum plasma concentration (C_max_), time to reach C_max_ (T_max_), area under the drug-time curve (AUC), elimination half-life (t_1/2_) and mean residence time (MRT) were calculated using DAS 3.0 software (Drug and Statistics, Shanghai, China). Concentrations below the lower limit of quantitation (BLOQ) were treated as zero for descriptive statistics and AUC calculation when occurring before T_max_; BLOQ values after T_max_ were excluded from terminal phase calculations. Not detected (ND) values in tissue distribution studies were assigned a value of half the LLOQ (10 ng/g) for descriptive purposes only; statistical comparisons were not performed for parameters where >50% of samples were ND. All data were expressed as mean ± standard deviation (SD). For sex-stratified tissue distribution analyses (*n* = 3 per sex per time point), no correction for multiple comparisons was applied due to the exploratory nature of these analyses. Findings should be interpreted as descriptive rather than confirmatory.

Pharmacokinetic parameters (AUC and C_max_) were log-transformed (natural logarithm) prior to statistical analysis. Normality of log-transformed PK parameters was assessed using the Shapiro–Wilk test. Homogeneity of variances was verified by Levene’s test. Statistical comparisons between two groups were performed using Student’s *t*-test on log-transformed AUC and C_max_ values. A value of *p* < 0.05 was considered statistically significant. Graphical representations were generated using Origin software 8.0 (OriginLab, Northampton, MA, USA).

## 4. Conclusions

A sensitive and reliable LC-MS/MS method was developed and fully validated for the simultaneous quantification of LY104 and its metabolite M1 in rat plasma, tissue homogenates, and excreta. The method was successfully applied to a comprehensive preclinical pharmacokinetic study of LY104 in rats. Following intravenous administration, M1 was rapidly formed in plasma. No sex-related differences were observed in the pharmacokinetic parameters or tissue distribution of LY104, and no significant accumulation occurred after seven daily doses under the present experimental conditions in rats. Tissue distribution studies confirmed that LY104 and M1 were widely distributed, with no significant sex differences observed at any time point. LY104 exhibited high protein binding (>99%) across all tested species, with no significant concentration or species differences. Species-dependent metabolism was observed in both plasma and liver microsomes. The total cumulative excretion of LY104 and M1 in urine and feces accounted for approximately 33% of the dose, indicating extensive metabolism and elimination primarily as metabolites. Notably, negligible brain penetration and preferential lung accumulation of LY104 address a major clinical limitation of existing PDE4 inhibitors (CNS-mediated emetic side effects) positioning LY104 as a promising candidate with an improved safety profile for respiratory disease therapy. Therefore, the robust analytical method and comprehensive ADME data provide essential support for the further development of LY104 as a therapeutic agent for COPD.

## Figures and Tables

**Table 1:** Intra- and inter-day precision and accuracy of LY104 and M1 in rat plasma (*n* = 6).

Analyte	Spiked (ng/mL)	Batch1 (Mean ± SD)	Batch2 (Mean ± SD)	Batch3 (Mean ± SD)	Overall Mean (ng/mL)	Intra-Day RSD (%)	Inter-Day RSD (%)	RE (%)
LY104	20	17.93 ± 1.33	18.49 ± 2.69	19.33 ± 1.34	18.58	7.44	9.34	−7.09
	40	41.70 ± 3.29	40.86 ± 1.06	39.66 ± 1.14	40.74	7.89	6.16	1.85
	400	425.20 ± 8.70	437.42 ± 11.83	424.79 ± 11.04	429.14	2.05	4.10	7.28
	1000	927.02 ± 13.98	991.18 ± 30.75	1024.68 ± 24.84	980.96	1.51	12.39	−1.90
M1	20	23.32 ± 2.10	23.20 ± 2.33	20.62 ± 0.88	22.38	9.00	16.72	11.90
	40	44.10 ± 2.24	44.24 ± 1.80	40.61 ± 3.05	42.98	5.09	11.74	7.46
	400	407.87 ± 17.90	417.50 ± 8.60	424.48 ± 9.19	416.62	4.39	4.90	4.15
	1000	972.64 ± 14.17	1031.12 ± 31.50	1064.65 ± 25.26	1022.80	1.46	11.15	2.28

**Table 2:** Dilution integrity of LY104 and M1 in rat plasma (*n* = 6).

Analyte	Spiked (ng/mL)	Dilution Factor	Mean ± SD (ng/mL)	RSD (%)	RE (%)
LY104	10,000	100	11,220.02 ± 84.19	0.75	12.20
M1	10,000	100	10,470.95 ± 430.11	4.11	4.71

**Table 3:** Recovery of LY104 and M1 in rat plasma (*n* = 3).

Analyte	Concentration (ng/mL)	Recovery	
		Mean ± SD (%)	RSD (%)
LY104	40	63.17 ± 3.62	5.73
	400	75.67 ± 2.57	3.39
	1000	83.35 ± 1.77	2.12
M1	40	85.66 ± 4.07	4.75
	400	94.32 ± 2.82	2.99
	1000	91.31 ± 0.65	0.72
BAEE	50	98.63 ± 2.20	2.23

**Table 4:** Matrix effects of LY104 and M1 in rat plasma (*n* = 18).

Analyte	Concentration (ng/mL)		Mean ± SD (ng/mL)	RSD (%)	RE (%)
LY104	LQC	40	39.01 ± 2.63	6.73	−2.47
	HQC	1000	975.20 ± 29.65	3.04	−2.48
M1	LQC	40	39.17 ± 2.31	5.89	−2.08
	HQC	1000	932.78 ± 36.07	3.87	−6.72

**Table 5:** Stability of LY104 and M1 in rat plasma under various storage conditions (*n* = 3).

Stability Conditions	Analyte	Concentration (ng/mL)	Mean ± SD (ng/mL)	RSD (%)	RE (%)
Ice for 20 min	LY104	40	37.03 ± 1.53	4.12	−7.42
		1000	1041.80 ± 42.86	4.11	4.18
	M1	40	43.22 ± 2.78	6.43	8.05
		1000	992.87 ± 38.43	3.87	−0.71
Room temperature for 6 h	LY104	40	40.57 ± 0.85	2.09	1.42
		1000	995.86 ± 2.90	0.29	−0.41
	M1	40	39.32 ± 1.03	2.61	−1.71
		1000	964.45 ± 16.85	1.75	−3.55
Long-term for 35 days (−80 °C)	LY104	40	40.95 ± 1.26	3.07	2.39
		1000	897.71 ± 44.80	4.99	−10.23
	M1	40	38.68 ± 3.18	8.22	−3.30
		1000	1127.37 ± 119.49	10.60	12.74
Three freeze–thaw cycles	LY104	40	39.45 ± 1.56	3.96	−1.38
		1000	853.84 ± 12.17	1.43	−14.62
	M1	40	38.24 ± 3.17	8.29	−4.39
		1000	1047.20 ± 28.08	2.68	4.72
Autosampler for 24 h (20 °C)	LY104	40	41.06 ± 1.72	4.18	2.64
		1000	984.52 ± 41.29	4.19	−1.55
	M1	40	38.56 ± 3.27	8.47	−3.59
		1000	1003.17 ± 40.66	4.00	0.32

**Table 6:** Pharmacokinetic parameters of M1 in rats following a single intravenous administration of LY104 at 0.2, 1, and 5 mg/kg (Mean ± SD; *n* = 12 for 0.2 and 1 mg/kg groups; *n* = 8 for 5 mg/kg group).

Parameters	0.2 mg/kg	1 mg/kg	5 mg/kg
AUC_0–t_ (μg/L·h)	202.49 ± 147.87	1789.05 ± 613.02	8936.27 ± 2650.15
AUC_0–∞_ (μg/L·h)	512.46 ± 138.89	2110.43 ± 594.38	10,720.74 ± 4330.54
t_1/2_ (h)	7.59 ± 3.53	4.90 ± 1.64	8.24 ± 4.05
Vz (L/kg)	256.51 ± 205.71	3.78 ± 1.99	5.80 ± 2.41
CLz (L/h/kg)	28.64 ± 22.76	0.52 ± 0.20	0.54 ± 0.22
MRT_0–t_ (h)	3.31 ± 1.07	5.89 ± 1.68	7.67 ± 1.17
T_max_ (h)	1.83 ± 1.11	2.29 ± 2.30	2.25 ± 1.49
C_max_ (μg/L)	41.54 ± 15.91	217.53 ± 74.89	953.88 ± 220.82

**Table 7:** Pharmacokinetic parameters of LY104 and its metabolite M1 in rats after the first and seventh intravenous doses of LY104 (1 mg/kg) (*n* = 12).

Parameters	LY104		M1	
	Day 1 ^†^	Day 7	Day 1	Day 7
AUC_0–t_ (μg/L·h)	101.86 ± 63.74	99.89 ± 44.24	1789.05 ± 613.02	3007.83 ± 1093.08 *
AUC_0–∞_ (μg/L·h)	124.48 ± 53.51	117.08 ± 47.97	2110.43 ± 594.38	3258.82 ± 1072.83 *
t_1/2_ (h)	0.43 ± 0.48	0.29 ± 0.14	4.90 ± 1.64	5.33 ± 1.04
Vz (L/kg)	4.65 ± 4.22	3.97 ± 2.29	3.78 ± 1.99	2.58 ± 0.85
CLz (L/h/kg)	9.05 ± 2.79	10.08 ± 4.49	0.52 ± 0.20	0.34 ± 0.13
MRT_0–t_ (h)	0.22 ± 0.28	0.17 ± 0.08	5.89 ± 1.68	6.58 ± 1.22
C_max_ (μg/L)	533.44 ± 344.69	584.02 ± 272.14	217.53 ± 74.89	324.33 ± 113.44 *

^†^ Data for LY104 on day 1 are from our previously published study [18]; all other data are reported here for the first time; * *p* < 0.05, *p*-values represent the differences between 1 day and 7 day; *p* values were calculated from *t*-test on log-transformed AUC and C_max_.

**Table 8:** B/P ratio of LY104 in rat blood (*n* = 3).

Concentration	B/P Ratio	Blood/Plasma Concentration Ratio	Blood Cell/Plasma Concentration Ratio
200	0.58 ± 0.04	0.78 ± 0.08	0.05 ± 0.09
1000	0.64 ± 0.03	0.67 ± 0.04	0.18 ± 0.06
5000	0.60 ± 0.01	1.07 ± 0.02	0.08 ± 0.02

**Table 9:** Plasma protein binding rates of LY104 in different species (*n* = 3).

Species	Plasma Protein Binding Rate (%)		
	200 ng/mL	1000 ng/mL	5000 ng/mL
Mouse	99.87 ± 0.08	99.85 ± 0.07	99.77 ± 0.10
Rat	>99.98	99.96 ± 0.02	99.80 ± 0.16
Dog	>99.98	99.95 ± 0.02	99.59 ± 0.24
HSA	>99.98	99.98 ± 0.01	99.93 ± 0.01

HSA: human serum albumin.

**Table 10:** In vitro metabolic stability of LY104 in rat, dog, and human plasma (*n* = 3).

System	Species	t_1/2_ (min)	CL (μL/min/mg)
Plasma	rat	0.72 ± 0.03	1927.48 ± 82.87
	dog	123.39 ± 93.70	23.30 ± 25.62
	human	0.69 ± 0.04	2021.57 ± 107.02
Liver microsomes	rat	40.44 ± 4.20	34.53 ± 3.81
	dog	8.97 ± 1.09	155.93 ± 17.64
	human	14.28 ± 2.35	98.92 ± 16.82

## Data Availability

Data will be made available on request from the corresponding author.

## Supplementary Materials

The following supporting information can be downloaded at: https://www.mdpi.com/article/10.3390/md24060215/s1, Table S1: The typical standard curves of LY104 and M1 (*n* = 3) in rat plasma; Figure S1: The typical MRM chromatograms of LY104, M1, and BAEE in rat liver samples: (A) blank liver sample; (B) blank liver sample spiked with LY104, M1, and BAEE at LLOQ; (C) liver samples collected at 3 h after intravenous administration of LY104 (1 mg/kg); Table S2: Regression equations of LY104 and M1 in rat tissue samples; Table S3: The precision and accuracy of LY104 and M1 in rat liver tissue; Figure S2: The typical MRM chromatograms of LY104, M1, and BAEE in rat urine samples: (A) blank urine sample; (B) blank urine sample spiked with LY104, M1, and BAEE at LLOQ; (C) urine samples collected at 4 h after intravenous administration of LY104 (1 mg/kg); Figure S3: The typical MRM chromatograms of LY104, M1, and BAEE in rat feces samples: (A) blank feces sample; (B) blank feces sample spiked with LY104, M1, and BAEE at LLOQ; (C) feces samples collected at 4 h after intravenous administration of LY104 (1 mg/kg); Table S4: Regression equations of LY104 and M1 in rat feces and urine samples; Table S5: Precision and accuracy of LY104 and M1 in rat urine and feces; Table S6: Sex-stratified pharmacokinetic parameters of LY104 and its metabolite M1 in rats. Following a single intravenous administration of LY104 at 0.2, 1, and 5 mg/kg, pharmacokinetic parameters were analyzed separately for male and female rats. This extends our previous report [18], which presented mixed-sex mean values (Mean ± SD; *n* = 12 for 0.2 and 1 mg/kg groups; *n* = 8 for 5 mg/kg group); Table S7: Sex-stratified pharmacokinetic parameters of LY104 and M1 in rats; Table S8: Sex comparison of LY104 tissue distribution in rats following a single intravenous administration of LY104 (1 mg/kg). Mean tissue concentrations at 0.5, 1, and 3 h post-dose are derived from our previously published data [18] but have been reanalyzed to reveal sex differences; data at 6 h are newly reported in this study (*n* = 3); Table S9: Mean concentrations of M1 in various tissues of rats at different time points following a single intravenous administration of LY104 (1 mg/kg). These tissue distribution data for M1 are reported here for the first time, with equal numbers of male and female rats (*n* = 3); Table S10: Pharmacokinetic parameters of LY104 in rat tissues after a single intravenous administration of LY104 (1 mg/kg) (*n* = 3); Table S11: Pharmacokinetic parameters of M1 in rat tissues after a single intravenous administration of LY104 (1 mg/kg) (*n* = 3).

## Abbreviations

The following abbreviations are used in this manuscript:

AUC	Area under the drug-time curve
BAEE	N-benzoyl-L-arginine ethyl ester hydrochloride
BBB	Blood–brain barrier
B/P	Blood-to-plasma
cAMP	Cyclic adenosine monophosphate
C_max_	Maximum plasma concentration
CNS	Central nervous system
COPD	Chronic obstructive pulmonary disease
DMSO	Dimethyl sulfoxide
ESI	Electrospray ionization
HQC	High-quality control
HSA	Human serum albumin
IS	Internal standard
ISR	Incurred sample reanalysis
LC-MS/MS	Liquid chromatography-tandem mass spectrometry
LLOQ	Lower limit of quantitation
LQC	Low-quality control
MQC	Medium-quality control
MRM	Multiple reaction monitoring
MRT	Mean residence time
NSB	Non-specific binding
PDE4	Phosphodiesterase 4
QC	Quality control
RE	Relative error
RSD	Relative standard deviation
SD	Standard deviation
S/N	Signal-to-noise ratio
SPF	Specific pathogen-free
t_1/2_	Elimination half-life
ULOQ	Upper limit of quantification
