# Supplementary material for: Comprehensive Pharmacokinetics of the Marine-Derived PDE4 Inhibitor LY104 and Its Major Metabolite M1 in Rats: A Validated LC-MS/MS Method with Sex Comparison, Multiple-Dose, Protein Binding, Metabolic Stability, and Excretion Studies
Source: Mar Drugs. 2026 Jun 15;24(6):215. doi: 10.3390/md24060215 (PMC13301581; doi:10.3390/md24060215)
Supplement: Supplementary file 1 [file marinedrugs-24-00215-s001.zip › marinedrugs-4335623-supplementary.pdf]

Supporting Information

Comprehensive Pharmacokinetics of the Marine-Derived PDE4 Inhibitor LY104 and Its Major Metabolite M1 in Rats: A Validated LC-MS/MS Method with Sex Comparison, Multiple-Dose, Protein Binding, Metabolic Stability, and Excretion Studies

Xiaochen Niu[1,†], Jun Zhao[1,†], Deqi Ding[2,†], Wei He[2] , Guanhua Du[1], Jiejie Hao[2,*], and Jianchun Zhao[1,*]

[1] *Marine Biomedical Research Institute of Qingdao, Ocean University of China, Qingdao 266073, China*

[2] *School of Medicine and Pharmacy, Ocean University of China, Qingdao 266003, China*

[†] These authors made equal contributions to this work.

***Correspondence:**
2009haojie@ouc.edu.cn (J.J., Hao); zhaojianchun@ouc.edu.cn (J.C., Zhao)

Contents:

- **Method validation**
- **Pharmacokinetic studies**
- **Tissue distribution**

1 Method validation

Table S1. The typical standard curves of LY104 and M1 (n = 3) in rat plasma.

Analyte	Batch	Intercept	Slope	r^2
	1	0.043343	0.255996	0.9920
LY104	2	0.063562	0.244228	0.9912
	3	0.033512	0.221725	0.9975
	1	0.000984	0.019957	0.9974
M1	2	0.001919	0.019314	0.9934
	3	0.001018	0.018191	0.9992

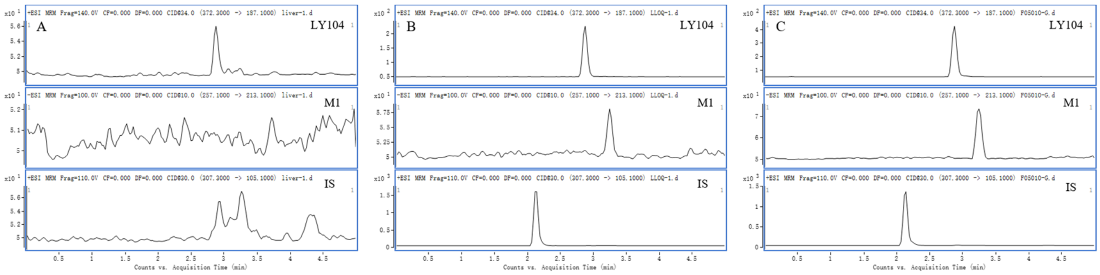

Figure S1. The typical MRM chromatograms of LY104, M1, and BAEE in rat liver samples: (A) blank liver sample; (B) blank liver sample spiked with LY104, M1, and BAEE at LLOQ; (C) liver samples collected at 3 h after intravenous administration of LY104 (1 mg/kg).

Table S2. Regression equations of LY104 and M1 in rat tissue samples.

Analyte	Tissue	Linear regression equation	r^2
	liver	y = 0.200999x + 0.013664	0.9968
	lung	y = 0.313099x + 0.027961	0.9900
	colon	y = 0.234515x + 0.034114	0.9913
LY104	brain	y = 0.275504x + 0.022089	0.9911
	kidney	y = 0.299217x + 0.026697	0.9935
	muscle	y = 0.259366x + 0.024490	0.9918
	stomach	y = 0.357317x + 0.033232	0.9919
	liver	y = 0.012700x + 0.001676	0.9988
	lung	y = 0.012946x + 0.001866	0.9960
	colon	y = 0.012889x + 0.000327	0.9970
M1	brain	y = 013981x – 0.000030	0.9985
	kidney	y = 0.013725x + 0.001528	0.9937
	muscle	y = 0.011253x + 0.001201	0.9950
	stomach	y = 0.018169x + 0.000619	0.9968

Table S3. The precision and accuracy of LY104 and M1 in rat liver tissue.

Analyte	QC (ng/mL)	Concentration (ng/mL)	RSD (%)	RE (%)
LY104	20	21.85 ± 1.29	5.90	9.27
	40	43.20 ± 2.20	5.09	7.99
	400	401.08 ± 3.53	0.88	0.27
	1,000	1,007.89 ± 35.60	3.53	0.79
M1	20	18.51 ± 1.76	9.52	-7.46
	40	39.67 ± 3.37	8.49	-0.82
	400	384.59 ± 6.33	1.65	-3.85
	1,000	930.54 ± 37.12	3.99	-6.95

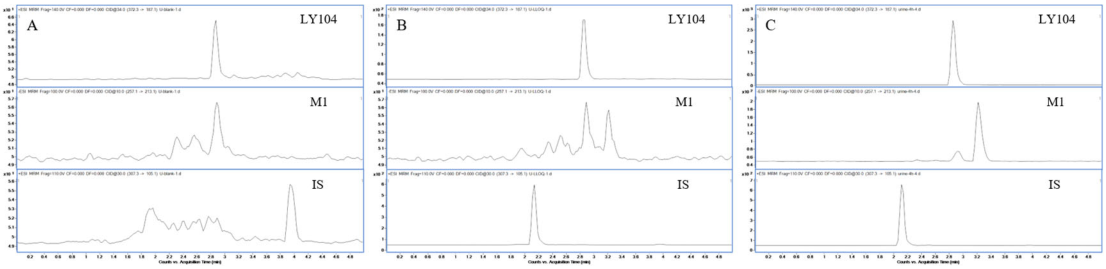

Figure S2. The typical MRM chromatograms of LY104, M1, and BAEE in rat urine samples: (A) blank urine sample; (B) blank urine sample spiked with LY104, M1, and BAEE at LLOQ; (C) urine samples collected at 4 h after intravenous administration of LY104 (1 mg/kg).

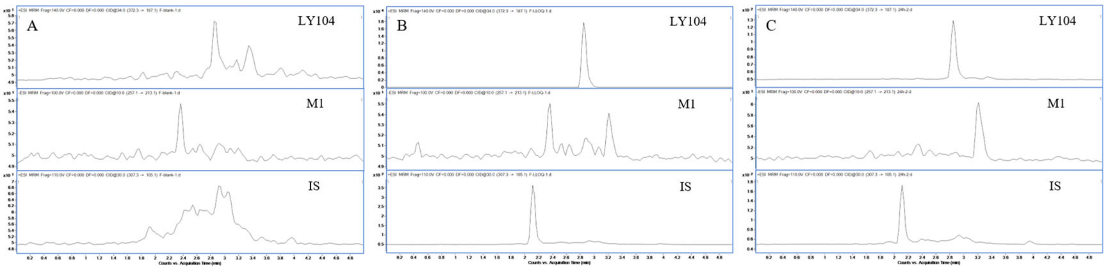

Figure S3. The typical MRM chromatograms of LY104, M1, and BAEE in rat feces samples: (A) blank feces sample; (B) blank feces sample spiked with LY104, M1, and BAEE at LLOQ; (C) feces samples collected at 4 h after intravenous administration of LY104 (1 mg/kg).

Table S4. Regression equations of LY104 and M1 in rat feces and urine samples.

Analyte	Matrix	Linear regression equation	r^2
LY104	feces	y = 0.457670x + 0.018384	0.9902
	urine	y = 0.623150x + 0.037479	0.9953
M1	feces	y = 0.024381x + 0.003130	0.9911
	urine	y = 0.038754x + 0.003487	0.9965

Table S5. Precision and accuracy of LY104 and M1 in rat urine and feces.

Analyte	QC (ng/mL)	Concentration (ng/mL)	RSD (%)	RE (%)	Concentration (ng/mL)	RSD (%)	RE (%)
		urine			feces		
LY104	40	40.33 ± 3.39	8.42	0.82	44.41 ± 6.34	14.28	11.03
	400	397.85 ± 22.68	5.70	-0.54	425.66 ± 16.42	3.86	6.42
	1,000	879.47 ± 38.54	4.38	-12.05	943.53 ± 81.29	8.62	-5.65
M1	40	42.45 ± 6.12	14.41	6.14	47.03 ± 4.58	9.73	17.57
	400	433.72 ± 28.67	6.61	8.43	448.56 ± 37.28	8.31	12.14
	1,000	1,009.30 ± 66.93	6.63	0.93	1,006.77 ± 77.62	7.71	0.68

2 Pharmacokinetic studies

Table S6. Sex-stratified pharmacokinetic parameters of LY104 and its metabolite M1 in rats. Following a single intravenous administration of LY104 at 0.2, 1, and 5 mg/kg, pharmacokinetic parameters were analyzed separately for male and female rats. This extends our previous report [1], which presented mixed-sex mean values (Mean ± SD; n=12 for 0.2 and 1 mg/kg groups; n=8 for 5 mg/kg group)

**Parameters**	**Sex**	**LY104**			**M1**		
		**0.2 mg/kg†**	**1 mg/kg†**	**5 mg/kg†**	**0.2 mg/kg**	**1 mg/kg**	**5 mg/kg**
AUC_{0-t} (μg/L·h)	male	17.52 ± 7.70	87.78 ± 56.31	550.69 ± 279.86	109.42 ± 73.72*	1,382.84 ± 486.08*	6,984.63 ± 1519.70*
	female	17.24 ± 8.26	115.94 ± 72.74	590.88 ± 59.68	295.56 ± 147.93*	2,195.25 ± 440.98*	10,887.91 ± 1980.17*
$AUC_{0-\infty}$ (μg/L·h)	male	18.85 ± 0.00	119.90 ± 38.37	579.11 ± 292.11	407.31 ± 52.24	1,638.41 ± 399.54*	7,915.75 ± 1991.15
	female	/	128.31 ± 67.16	610.81 ± 57.61	565.03 ± 140.79	2,582.45 ± 287.88*	13,525.74 ± 4337.33
$t_{1/2}$ (h)	male	/	0.34 ± 0.34	1.04 ± 0.76	6.33 ± 1.17	5.30 ± 1.43	7.67 ± 3.00
	female	/	0.50 ± 0.59	0.82 ± 0.47	8.22 ± 4.24	4.49 ± 1.86	8.81 ± 5.34
Vz (L/kg)	male	/	4.74 ± 5.54	12.80 ± 4.53	449.74 ± 72.47	4.99 ± 1.96*	7.16 ± 2.58
	female	/	4.57 ± 3.33	9.56 ± 4.95	159.89 ± 178.89	2.57 ± 1.16*	4.44 ± 1.40
CLz (L/h/kg)	male	10.61 ± 0.00	9.03 ± 2.74	10.88 ± 6.54	49.66 ± 6.47	0.65 ± 0.22*	0.67 ± 0.20
	female	/	9.07 ± 3.10	8.24 ± 0.77	18.13 ± 20.36	0.39 ± 0.05*	0.40 ± 0.15
MRT_{0-t}(h)	male	0.28 ± 0.00	0.13 ± 0.09	0.73 ± 0.60	2.16 ± 0.52**	5.90 ± 1.94	7.45 ± 1.01
	female	/	0.29 ± 0.36	0.42 ± 0.12	3.88 ± 0.74**	5.89 ± 1.57	7.90 ± 1.43
T_{max} (h)	male	0.11 ± 0.11	0.03 ± 0.00	0.09 ± 0.11	1.50 ± 0.55	1.42 ± 0.66	2.75 ± 1.50
	female	0.05 ± 0.03	0.08 ± 0.09	0.03 ± 0.00	2.17 ± 1.47	3.17 ± 3.06	1.75 ± 1.50
C_{max} (ng/mL)	male	147.68 ± 106.40	587.03 ± 385.30	2,029.20 ± 1511.63	36.29 ± 14.03	175.94 ± 45.02	903.07 ± 115.72
	female	172.83 ± 107.53	479.86 ± 325.63	3,230.05 ± 481.27	46.78 ± 17.15	259.11 ± 78.50	1,004.69 ± 305.78

† Data for LY104 are from our previous study and have been re-analyzed with sex stratification; * $P < 0.05$, *P-values* represent the sex differences between males and females; "/": Data not calculated; *P* values were calculated from t-test on log-transformed AUC and C_{max}.

Table S7. Sex-stratified pharmacokinetic parameters of LY104 and M1 in rats.

Analyte	Parameter	Dose	Sex	Mean ± SD	95% CI	*P* value	Ratio (M/F)	95% CI of ratio
LY104	AUC_{0-t} (μg/L·h)	0.2 mg/kg	M	17.52 ± 7.70	(8.74, 28.22)	$P > 0.05$	1.02	(0.59, 1.74)
			F	17.24 ± 8.26	(8.92, 26.89)			
		1 mg/kg	M	87.78 ± 56.31	(29.12, 163.99)	$P > 0.05$	0.76	(0.32, 1.40)
			F	115.94 ± 72.74	(61.72, 172.01)			
		5 mg/kg	M	550.69 ± 279.86	(188.57, 1,267.61)	$P > 0.05$	0.93	（0.32，2.13）
			F	590.88 ± 59.68	(503.00, 688.96)			
	C_{max} (ng/mL)	0.2 mg/kg	M	147.68 ± 106.40	(46.03, 278.49)	$P > 0.05$	0.85	（0.41，1.60）
			F	172.83 ± 107.53	(63.37, 311.02)			
		1 mg/kg	M	587.03 ± 385.30	(147.42, 1,243.33)	$P > 0.05$	1.22	（0.24，8.53）
			F	479.86 ± 325.63	(73.85, 1,224.36)			
		5 mg/kg	M	2,029.20 ± 1,511.63	(209.68, 9,161.82)	$P > 0.05$	0.63	（0.05，3.54）
			F	3,230.05 ± 481.27	(2,512.42, 4,081.99)			
M1	AUC_{0-t} (μg/L·h)	0.2 mg/kg	M	109.42 ± 73.72	(34.18, 209.36)	$P < 0.05$	0.37	（0.19，0.51）
			F	295.56 ± 147.93	(163.10, 442.29)			
		1 mg/kg	M	1,382.84 ± 486.08	(878.34, 1,944.77)	$P < 0.05$	0.63	（0.38，0.96）
			F	2,195.25 ± 440.98	(1,722.31, 2,690.06)			
		5 mg/kg	M	6,984.63 ± 1,519.71	(4,648.69, 10,071.15)	$P < 0.05$	0.64	（0.56，0.73）
			F	10,887.91 ± 1,980.17	(7,835.19, 14,715.26)			
	C_{max} (ng/mL)	0.2 mg/kg	M	36.29 ± 14.03	(21.84, 52.49)	$P > 0.05$	0.78	（0.52，1.15）
			F	46.78 ± 17.15	(28.86, 66.89)			
		1 mg/kg	M	175.94 ± 45.02	(120.77, 237.94)	$P > 0.05$	0.68	（0.42，1.09）
			F	259.11 ± 78.50	(180.80, 343.69)			
		5 mg/kg	M	903.07 ± 115.72	(733.46, 1,098.46)	$P > 0.05$	0.90	（0.70，1.23）
			F	1,004.69 ± 305.78	(596.49, 1,577.74)			

95% confidence intervals (CI) were calculated based on log-transformed AUC and C_{max} values using the geometric mean method. The male-to-female geometric mean ratio and its 95% CI were obtained by exponentiating the difference of the log-transformed means and its CI. $P < 0.05$: The 95% CI of the ratio does not include 1, corresponding to a statistically significant difference.

3 Tissue distribution

Table S8. Sex comparison of LY104 tissue distribution in rats following a single intravenous administration of LY104 (1 mg/kg). Mean tissue concentrations at 0.5, 1, and 3 h post-dose are derived from our previously published data [1] but have been reanalyzed to reveal sex differences; data at 6 h are newly reported in this study. (n = 3).

Organ		LY104（ng/mL or ng/g，Mean ± SD）			
		**0.5 h†**	**1 h†**	**3 h†**	**6 h**
Plasma	male	44.03 ± 9.82	23.39 ± 0.00	BLOQ	BLOQ
	female	32.99 ± 0.00	BLOQ	BLOQ	BLOQ
Liver	male	151.58 ± 76.25	43.23 ± 9.59	19.09 ± 2.14	BLOQ
	female	126.74 ± 37.39	133.07 ± 74.01	34.17 ± 10.65	25.06 ± 7.20
Heart	male	910.83 ± 398.84	1,164.61 ± 385.60	987.71 ± 271.40	659.53 ± 187.57
	female	1,275.23 ± 378.17	1,303.34 ± 295.92	886.17 ± 198.59	579.39 ± 121.83
Spleen	male	210.55 ± 64.17	158.00 ± 43.43	46.94 ± 23.05	19.58 ± 0.00
	female	224.41 ± 27.19	231.66 ± 124.30	39.52 ± 8.61	19.13 ± 0.00
Lung	male	689.32 ± 402.42	413.93 ± 38.49	170.47 ± 40.98	80.48 ± 18.55
	female	726.45 ± 68.72	505.37 ± 136.22	207.24 ± 64.60	76.35 ± 13.85
Colon	male	513.05 ± 93.76	562.81 ± 192.28	173.96 ± 43.72	64.36 ± 42.54
	female	452.59 ± 25.56	397.95 ± 55.06	183.96 ± 52.89	57.53 ± 5.78
Small intestine	male	979.33 ± 487.43	319.77 ± 51.56	195.98 ± 66.93	24.53 ± 11.95
	female	482.94 ± 71.18	813.24 ± 458.16	179.76 ± 8.11	43.00 ± 9.61
Kidney	male	4,145.15 ± 851.65*	1,764.62 ± 628.50	900.13 ± 527.01	198.19 ± 35.96
	female	2,529.30 ± 376.94*	1,911.33 ± 1180.75	737.61 ± 161.85	204.50 ± 15.49
Stomach	male	237.15 ± 46.41	297.95 ± 212.26	181.75 ± 147.21	29.10 ± 21.41
	female	128.99 ± 80.02	334.92 ± 93.04	160.45 ± 44.35	43.49 ± 5.68
Brain	male	ND	ND	ND	ND
	female	ND	ND	ND	ND
Fat	male	174.32 ± 123.59	76.89 ± 41.30	74.41 ± 21.93	51.41 ± 0.00
	female	112.82 ± 81.88	103.59 ± 37.03	35.48 ± 0.00	ND
Testis	male	25.90 ± 8.52	29.24 ± 0.00	27.30 ± 0.00	BLOQ
	female	/	/	/	/
Ovary	male	/	/	/	/
	female	440.42 ± 77.37	430.23 ± 145.57	168.70 ± 28.05	70.42 ± 11.39
Muscle	male	178.56 ± 30.37	157.83 ± 33.00	125.54 ± 27.90	162.82 ± 11.65
	female	211.41 ± 45.03	153.51 ± 32.40	157.37 ± 10.66	133.78 ± 35.34
Trachea	male	804.73 ± 128.01	547.12 ± 150.22	327.22 ± 86.20	255.14 ± 30.09*
	female	1254.41 ± 384.00	735.13 ± 282.48	366.11 ± 105.14	130.47 ± 19.49*

*: $P < 0.05$, *P-values* represent the sex differences between males and females; “/” : Data not calculated; “ND” : Not detected; BLOQ: Below the limit of quantification; † Data at 0.5, 1, and 3 h are sourced from our previously published research [1]; tissue concentrations at 6 h are reported here for the first time. This study also represents the first detailed sex stratification of LY104

tissue distribution at all time points.

Table S9. Mean concentrations of M1 in various tissues of rats at different time points following a single intravenous administration of LY104 (1 mg/kg). These tissue distribution data for M1 are reported here for the first time, with equal numbers of male and female rats (n = 3).

**Organ**		**M1（ng/mL，ng/g，Mean ± SD）**			
		**0.5 h**	**1 h**	**3 h**	**6 h**
Plasma	male	206.72 ± 48.50	207.33 ± 30.83*	124.85 ± 12.62*	89.05 ± 3.03*
	female	247.10 ± 10.81	305.41 ± 38.19*	219.13 ± 30.44*	144.01 ± 31.28*
Liver	male	164.79 ± 46.59	300.62 ± 101.97	270.56 ± 90.35	190.82 ± 20.53
	female	190.77 ± 50.44	285.77 ± 96.94	339.28 ± 46.21	221.86 ± 33.60
Heart	male	43.35 ± 4.65	48.00 ± 8.99	58.82 ± 20.17	35.02 ± 0.00
	female	32.78 ± 6.80	49.49 ± 16.70	53.74 ± 26.92	28.36 ± 5.83
Spleen	male	BLOQ	34.15 ± 1.19	28.03 ± 7.36	BLOQ
	female	20.22 ± 0.25	44.35 ± 31.07	34.90 ± 20.30	BLOQ
Lung	male	58.66 ± 33.65	52.17 ± 8.74	72.63 ± 19.85	43.21 ± 17.08
	female	58.17 ± 7.72	71.50 ± 22.34	80.22 ± 9.03	68.01 ± 10.51
Colon	male	34.69 ± 8.92	74.04 ± 42.78	47.48 ± 9.32	30.25 ± 3.25
	female	50.06 ± 9.82	49.94 ± 16.16	51.60 ± 7.72	46.82 ± 22.58
Small intestine	male	115.75 ± 116.43	77.88 ± 40.11	66.39 ± 38.81	78.54 ± 31.09
	female	44.05 ± 16.50	165.71 ± 79.74	69.27 ± 21.97	65.82 ± 43.55
Kidney	male	223.01 ± 117.18	188.34 ± 69.85	186.94 ± 51.33	119.02 ± 49.50
	female	179.89 ± 17.18	327.61 ± 183.26	211.86 ± 55.25	97.17 ± 33.27
Stomach	male	22.13 ± 10.36	32.89 ± 16.11	32.57 ± 15.53	13.25 ± 10.69
	female	21.01 ± 10.89	38.09 ± 6.30	38.87 ± 8.58	28.30 ± 9.48
Brain	male	BLOQ	BLOQ	BLOQ	BLOQ
	female	BLOQ	BLOQ	BLOQ	BLOQ
Fat	male	57.63 ± 0.00	26.93 ± 7.51	32.79 ± 15.15	19.90 ± 4.03
	female	37.66 ± 22.25	22.84 ± 11.50	25.81 ± 17.49	24.30 ± 0.00
Testis	male	17.48 ± 0.35	21.62 ± 1.71	47.62 ± 23.28	27.89 ± 3.49
	female	/	/	/	/
Ovary	male	/	/	/	/
	female	38.05 ± 15.67	50.02 ± 30.72	68.71 ± 26.05	35.55 ± 10.81
Muscle	male	BLOQ	BLOQ	BLOQ	BLOQ
	female	BLOQ	BLOQ	BLOQ	BLOQ
Air tube	male	90.44 ± 23.82	89.61 ± 44.26	124.65 ± 63.67	96.49 ± 23.19
	female	94.57 ± 35.83	103.05 ± 64.60	149.88 ± 50.11	142.23 ± 40.32

*P < 0.05, *P-values* represent the sex differences between males and females; "/": Data not calculated; BLOQ: Below the limit of quantification.

Table S10. Pharmacokinetic parameters of LY104 in rat tissues after a single intravenous administration of LY104 (1 mg/kg) (n = 3).

Organ	$AUC_{0\text{-}t}$ (μg/L·h)	$AUC_{0\text{-}\infty}$ (μg/L·h)	$t_{1/2}$ (h)	T_{max} (h)	C_{max} (μg/L)	Kp
Plasma	15.49 ± 24.86	/	/	/	41.27 ± 9.74	1.00
Heart	5,625.76 ± 651.86	11,851.42 ± 4,075.72	6.69 ± 3.13	1.25 ± 0.88	1,347.73 ± 224.09	363.19
Liver	356.32 ± 165.14	484.39 ± 73.65	2.79 ± 1.12	0.58 ± 0.20	147.35 ± 61.88	23.00
Spleen	499.09 ± 134.50	664.82 ± 175.10	1.61 ± 0.00	0.67 ± 0.26	248.72 ± 71.67	32.22
Lung	1,833.34 ± 341.08	2034.31 ± 255.23	1.91 ± 0.87	0.58 ± 0.20	737.94 ± 201.39	118.36
trachea	2,902.13 ± 692.19	3,256.59 ± 792.06	1.90 ± 0.59	0.50 ±0.00	1,029.57 ± 355.25	187.36
Kidney	8,343.06 ± 1,592.85*	8,732.90 ± 1,555.89*	1.40 ± 0.17	0.58 ± 0.20	3,525.65 ± 883.82	538.61
Stomach	1,005.72 ± 237.39	1,102.12 ± 246.40	1.76 ± 0.42	1.33 ± 0.82	356.56 ± 108.48	64.93
small intestine	2,040.25 ± 640.62	2,108.19 ± 626.81	1.32 ± 0.27	0.67 ± 0.26	914.95 ± 405.93	131.71
Colon	1,511.19 ± 226.42	1,674.23 ± 285.72	1.74 ± 0.33	0.75 ± 0.27	540.30 ± 105.95	97.56
Muscle	930.39 ± 115.12	2,220.83 ± 1,245.86	7.67 ± 4.74	0.58 ± 0.20	196.89 ± 36.93	60.06
Brain	/	/	/	/	/	0.00
Ovary	709.43 ± 777.63	1,648.46 ± 62.45	2.27 ± 0.23	0.67 ± 0.29	514.33 ± 74.76	45.80
Testis	18.31 ± 35.70	/	/	/	27.01 ± 6.33	1.18
Fat	290.28 ± 249.57	1,186.61 ± 0.00	6.01 ± 0.00	0.75 ± 0.27	163.02 ± 85.53	18.74

Tissue-plasma partition coefficients (Kp) was calculated using $AUC_{0\text{-}t}$ tissue/$AUC_{0\text{-}t}$ plasma;

$^{*}P < 0.05$, *P-values* represent the sex differences between males and females; "/": Data not calculated. *P* values were calculated from t-test on log-transformed AUC and C_{max}.

Table S11. Pharmacokinetic parameters of M1 in rat tissues after a single intravenous administration of LY104 (1 mg/kg) (n = 3).

Organ	AUC_{0-t} (μg/L·h)	$AUC_{0-\infty}$ (μg/L·h)	$t_{1/2}$ (h)	T_{max} (h)	C_{max} (μg/L)	Kp
Plasma	1,090.11 ± 271.32*	1,976.42 ± 783.92*	5.08 ± 1.85	0.83 ± 0.26	261.32 ± 60.88	1.00
Heart	237.70 ± 85.33	646.35 ± 432.98	8.03 ± 6.98	2.33 ± 1.03	61.72 ± 14.37	0.22
Liver	1,557.19 ± 260.62	5,387.10 ± 4,295.70	15.15 ± 11.67	1.58 ± 1.11	338.25 ± 76.97	1.43
Spleen	57.82 ± 35.88	/	/	1.67 ± 1.03	38.18 ± 16.94	0.05
Lung	398.68 ± 74.88	1,215.29 ± 1,496.75	15.46 ± 15.43	2.75 ± 1.94	80.98 ± 15.26	0.37
trachea	725.35 ± 171.20	5,510.70 ± 6,482.56*	56.01 ± 29.39	2.42 ± 2.06	161.64 ± 49.08	0.67
Kidney	1,142.07 ± 208.24	2,028.43 ± 703.61	5.12 ± 2.98	1.58 ± 1.11	309.54 ± 123.39	1.05
Stomach	180.12 ± 44.21	517.28 ± 531.64	9.70 ± 9.12	2.00 ± 1.10	41.37 ± 10.74	0.17
small intestine	531.32 ± 83.79	1,853.09 ± 2,683.07	12.70 ± 20.58	1.25 ± 0.88	161.84 ± 71.51	0.49
Colon	290.60 ± 43.38	622.09 ± 138.45	7.11 ± 3.09	2.17 ± 2.04	69.83 ± 27.40*	0.27
Muscle	/	/	/	/	/	/
Brain	/	/	/	/	/	/
Ovary	319.21 ± 62.64	4,058.98 ± 5,733.69	48.35 ± 70.42	2.33 ± 1.16	83.81 ± 4.72	0.29
Testis	169.81 ± 98.44	218.46 ± 71.96	/	3.00 ± 0.00	47.62 ± 23.28	0.16
Fat	129.41 ± 51.48	252.32 ± 76.90	3.32 ± 2.28	1.42 ± 1.24	45.78 ± 14.05	0.12

Tissue-plasma partition coefficients (Kp) was calculated using AUC_{0-t} tissue/AUC_{0-t} plasma;

*$P < 0.05$, *P-values* represent the sex differences between males and females; "/": Data not calculated. *P* values were calculated from t-test on log-transformed AUC and C_{max}.

Reference

1. Dai, X.; Hao, J.; Zhang, Y.; Yang, Y.; Meng, W.; Lu, F.; Zhao, J.; Du, G.; Wan, S.; Hao, J. Discovery of Marine-Inspired Guanidine-Based PDE4 Inhibitors for the Treatment of Chronic Obstructive Pulmonary Disease. *Mar. Drugs* **2026**, *24*, 90.
